# Two-Year Switzerland Cohort Results from a Global Observational Study Investigating Proactive Dosing with Intravitreal Aflibercept 2 mg in Neovascular Age-Related Macular Degeneration

**DOI:** 10.3390/jcm14072370

**Published:** 2025-03-29

**Authors:** Katja Hatz, Aude Ambresin, Martin Schmid, Christian Prünte, Daniel Barthelmes, Tobias Machewitz, Helmut Allmeier, Gabor Mark Somfai

**Affiliations:** 1 Vista Eye Clinic Binningen, Hauptstrasse 55, 4102 Binningen, Switzerland; 2 Faculty of Medicine, University of Basel, 4056 Basel, Switzerland; 3 Swiss Visio Montchoisi, 1006 Lausanne, Switzerland; aambresin@swissvisio.net; 4 RétinElysée, Ophthalmology Center, 1006 Lausanne, Switzerland; 5 Eye Clinic, Lucerne Cantonal Hospital, 6000 Lucerne, Switzerland; martin.schmid@luks.ch; 6 Faculty of Health Sciences and Medicine, University of Lucerne, 6000 Lucerne, Switzerland; 7 Department of Ophthalmology, University Eye Clinic Basel, 4031 Basel, Switzerland; christian@pruente.ch; 8 Institute of Molecular and Clinical Ophthalmology Basel (IOB), 4031 Basel, Switzerland; 9 Vue. Eye Center, 2502 Biel/Bienne, Switzerland; 10 Department of Ophthalmology, University Hospital Zurich, 8091 Zurich, Switzerland; daniel.barthelmes@usz.ch (D.B.); gabor.somfai@stadtspital.ch (G.M.S.); 11 Department of Ophthalmology, University of Zurich, 8006 Zurich, Switzerland; 12 Bayer AG, 10115 Berlin, Germany; 13 Bayer Consumer Care, AG, 4002 Basel, Switzerland; helmut.allmeier@bayer.com; 14 Eye Department, Spross Research Institute, 8063 Zurich, Switzerland

**Keywords:** anti-vascular endothelial growth factor, clinical trial, intravitreal aflibercept, neovascular age-related macular degeneration, observational, real-world evidence, retina, Switzerland, vision

## Abstract

**Background/Objectives**: XTEND is the largest global, prospective, observational study of treatment-naïve patients with neovascular age-related macular degeneration (nAMD) receiving 2 mg of intravitreal aflibercept (IVT-AFL) in routine clinical practice designed to examine the real-world effectiveness of IVT-AFL proactive treatment regimens. The outcomes from the Switzerland cohort are reported here. **Methods**: Patients aged ≥50 years were eligible if they planned to receive IVT-AFL 2 mg. After three initial monthly IVT-AFL injections, treatment intervals could be extended (4-week minimum treatment interval). Visual and anatomic outcomes, treatment exposure, and safety were assessed. Statistics were descriptive. **Results**: Fifty-one patients were treated. At baseline, the mean ± standard deviation (SD) best-corrected visual acuity (BCVA) was 64.9 ± 17.9 letters, and central subfield thickness (CST) was 402 ± 106 µm. At month (M) 12 and M24, the mean (95% confidence interval [CI]) change from baseline in BCVA was +5.7 (1.9, 9.4) and +5.6 (1.3, 9.8) letters, respectively. In patients with a high baseline BCVA (≥70 letters [*n* = 28; mean ± SD: 77.5 ± 4.8 letters]), BCVA was maintained at ≥70 letters at M12 and M24 (mean change from baseline [range] +1.0 [−15.0, 11.0] and +1.1 [–10.0, 14.0], respectively). At M12 and M24, the mean (95% CI) change in CST was −125 (−161, −90) µm and −127 (−162, −93) µm, respectively. Patients received a mean ± SD of 9.5 ± 3.2 and 13.7 ± 6.0 injections by M12 and M24, respectively. No safety concerns were identified. **Conclusions**: In Swiss routine clinical practice, functional and anatomic improvements were achieved with IVT-AFL 2 mg proactive treatment in patients with nAMD over 24 months despite a relatively high baseline BCVA.

## 1. Introduction

Neovascular age-related macular degeneration (nAMD) is the most common cause of blindness in people older than 60 years, with a high prevalence in Europe [1]. In 2020, there were approximately 550,000 people living with vision loss in Switzerland, of which 20,000 were blind [2]. Intravitreal aflibercept (IVT-AFL) and ranibizumab are anti-vascular endothelial growth factor (anti-VEGF) therapies that have been shown to be effective treatment options for patients with nAMD, demonstrating robust vision improvements in randomized clinical trials (RCTs) [3,4,5,6,7,8]. However, optimal outcomes are not always observed outside of an RCT setting, with patients receiving fewer anti-VEGF injections and achieving lower visual acuity (VA) gains in clinical practice than those observed in RCTs [9,10].

Real-world evidence (RWE) can complement data from RCTs by providing insights into effectiveness, safety, and treatment patterns in real-world heterogenous patient populations based on locally defined treatment practices [11,12,13,14] and help to explain discrepancies between the outcomes observed in RCTs and routine clinical practice. The effectiveness of IVT-AFL to treat nAMD in routine clinical practice has been examined through the following multicenter, single-country studies: RAINBOW in France [15,16,17], PERSEUS in Germany [18], and PERSEUS-IT in Italy [19]. Although there are some RWE data demonstrating the effectiveness of IVT-AFL in Switzerland [20,21,22,23,24], there is a lack of fully prospective studies (previous studies were retrospective, with the exception of ASTERIA [20], which was both retrospective and prospective).

Ongoing real-world monitoring of the effectiveness and safety profile of IVT-AFL 2 mg continues to be relevant for the long-term use of IVT-AFL 2 mg, especially for patients in countries with restricted access to or no authorization to use high-dose anti-VEGF therapies. XTEND (eXtended and proacTive dosing regimEn in treatment-Naïve patients with neovascular age-related macular Degeneration; NCT03939767) was a 36-month, multicenter, prospective, observational study examining the effectiveness of proactive IVT-AFL treatment regimens (fixed dosing or treat-and-extend [T&E] regimens) in routine clinical practice in treatment-naïve patients with nAMD [25]. The study was conducted in 17 countries globally and is the first fully prospective study of real-world proactive IVT-AFL treatment regimens in treatment-naïve patients with nAMD in Switzerland. As the coronavirus disease 2019 (COVID-19) pandemic took place during the study period, there was a unique opportunity to gain insight into modifications to real-world clinical practice. This article presents the 12- and 24-month effectiveness and safety outcomes in the XTEND Switzerland cohort and discusses the findings in the Switzerland cohort in the context of the XTEND Global cohort [25]. There was no pre-defined hypothesis; the analyses presented here are explorative and descriptive.

## 2. Materials and Methods

### 2.1. Study Design

XTEND (NCT03939767) was a multinational, multicenter, prospective, observational study designed to examine the effectiveness of real-world proactive IVT-AFL treatment regimens in treatment-naïve patients with nAMD. Countries with European Medicines Agency (EMA)-aligned labels (Argentina, Belgium, Mainland China, Colombia, Denmark, France, Ireland, Italy, Norway, South Korea, Spain, Sweden, Thailand, and the UK) and non-EMA-aligned labels (Australia, Canada, and Switzerland) were included. The study enrolled patients between May 2019 and May 2020, with follow-up for up to 36 months.

### 2.2. Compliance with Ethics Guidelines

XTEND was carried out in accordance with EMA guidelines and recommendations and applicable local law(s) and regulation(s). The protocol and any amendments were reviewed and approved by each site’s independent ethics committee or institutional review board prior to the study’s commencement (Supplementary Materials Table S1).

The study was performed in accordance with the Helsinki Declaration of 1964. The recommendations of the European Federation of Pharmaceutical Industries and Associations, European Network of Centers for Pharmacoepidemiology and Pharmacovigilance, Good Pharmacovigilance Practices (GVP module VI), and International Council for Harmonization Guideline E3: Good Clinical Practice were also followed wherever possible. Written informed consent was provided by all patients before the start of data collection. The reporting of results was per “Strengthening the reporting of observational studies in epidemiology” (STROBE) guidance”; this is available as Supplementary Materials.

### 2.3. Patient Population

Treatment-naïve patients aged ≥50 years with a diagnosis of nAMD were eligible for enrollment into XTEND. Patients were not eligible if they received prior therapy for nAMD (anti-VEGF or other conditions) in the eye under study. The decision to initiate treatment with IVT-AFL in a proactive regimen had to be made for the patient as part of routine clinical practice by the treating physician and according to the local label. Patients were enrolled in the study after the decision to initiate IVT-AFL treatment had been made. Exclusion criteria included the following: participation in an investigational program with interventions outside of routine clinical practice; contraindications to IVT-AFL; planned treatment outside of local marketing authorization; eye diseases (e.g., advanced glaucoma or visually significant cataracts) that were likely to require surgery in the eye under study during the observation period; the concomitant ocular or systemic administration of drugs up to 3 months before IVT-AFL treatment that could interfere with or potentiate IVT-AFL treatment; and retinal diseases that might interfere with the treatment of nAMD. One eye per patient was included in the study. For participants who fulfilled the inclusion/exclusion criteria for both eyes, the eye with the worse baseline VA was included in the study.

### 2.4. Interventions

IVT-AFL 2 mg was given to patients in proactive regimens (fixed dosing or T&E) according to the local label and standard of care and the discretion of the treating physician. According to the Swiss label, after 3 initial monthly injections, treatment could be continued with 8 weekly injections (fixed dosing), or treatment could be extended by a maximum of 4 weekly increments to a maximum injection interval of 16 weeks (T&E) [26]. The majority of countries in the XTEND Global cohort followed the EMA label, whereby after 3 initial monthly injections, the treatment of patients could be extended by 2 or 4 weekly treatment increments. The minimum treatment interval was 8 weeks during the first 12 months of treatment, and the maximum treatment interval was 16 weeks [25,27]. Treatment, treatment extension, and monitoring decisions were made at the discretion of the treating physician; the protocol did not define the dates for treatment, treatment intervals for extension, and follow-up visits. The baseline visit was recorded by the treating physician and was defined as the day of the first IVT-AFL injection.

Alterations to the planned proactive treatment regimens might have occurred due to the COVID-19 pandemic. The Board of the Swiss Vitreoretinal Group recommended that patients on T&E regimens continue treatment, with a pause to the treatment interval extension and diagnostic tests [28]. These recommendations were not mandatory but may have been adopted by many Swiss clinics. In clinics that continued with planned treatment regimens, patients may have been unable or unwilling to attend appointments due to the COVID-19 pandemic.

### 2.5. Study Endpoints

The primary endpoint was the mean change in best-corrected visual acuity (BCVA) from baseline to 12 months, measured by the Early Treatment Diabetic Retinopathy Study (ETDRS) letters or Snellen chart with conversion to ETDRS [29]. Secondary endpoints at 12 and 24 months included the following: the change in BCVA from baseline to 24 months; the proportion of patients (eyes) gaining or losing VA compared with baseline, with letter scores of ≥0 letters, ≥5 letters, ≥10 letters, and ≥15 letters; the proportion of patients (eyes) achieving a Snellen equivalent of 20/40 or better; changes in central subfield thickness (CST) from baseline; the injection number; and injection intervals.

Safety was assessed throughout the study. The Medical Dictionary for Regulatory Activities was used to report and code all adverse events (AEs). Treatment-emergent AEs (TEAEs) were defined as those occurring within 30 days of the patient’s last IVT-AFL injection.

### 2.6. Statistical Analysis

Statistics are descriptive and explorative. Categorical variables are described using frequency tables. Continuous variables are described by sample statistics (e.g., mean and standard deviation [SD]), and an absolute value and change from the baseline are given for each time point, if applicable. To describe the effectiveness of the IVT-AFL treatment regimen, the mean change in BCVA from baseline was calculated, and 95% confidence intervals (CIs) were provided. The full analysis set (FAS) included patients who received ≥1 injection of IVT-AFL, ≥1 VA measurement with an available VA letter score at baseline, and ≥1 post-baseline assessment of VA measured ≥5 days after an IVT-AFL injection. VA and CST outcomes were evaluated at baseline and monthly until month 24. For time points for month 12 and month 24, the data for each patient were assessed from visits within 300 and 420 days (month 12) and 660 and 780 days (month 24). For missing data in BCVA or CST, the last observation was carried forward (LOCF). There were no time limits defined for the LOCF. The safety analysis set (SAS) included all patients who received ≥1 injection of IVT-AFL 2 mg. Safety analyses were performed on TEAEs; other AEs were tabulated without further analysis.

## 3. Results

### 3.1. Patients

**Figure 1:**
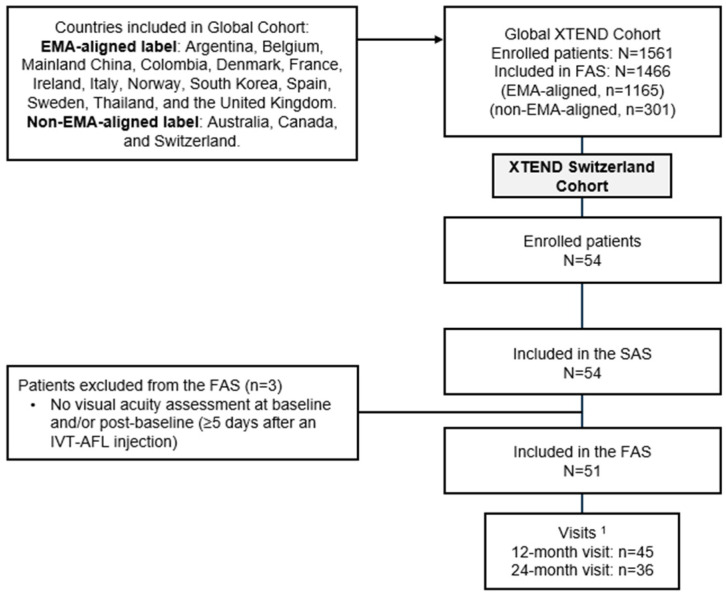
Patient disposition. ^1^ Patients with a visit within the 12-month (360 ± 60 days) or 24-month (720 ± 60 days) visit window. FAS completers were defined as patients with valid visual acuity assessments at baseline and at Month 12 (360 ± 60 days). By Month 24 (720 ± 60 days), 20 patients had withdrawn from the study and did not have a valid visual acuity assessment at Month 24. EMA: European Medicines Agency; FAS: full analysis set; IVT-AFL: intravitreal aflibercept; SAS: safety analysis set.

At the time of the 24-month analysis, 1561 patients from 127 centers had been enrolled in XTEND (Figure 1). Of these, 54 patients were part of the XTEND Switzerland cohort. All patients in the Switzerland cohort were included in the SAS, and 51 patients (94.4%) were included in the FAS (no baseline and/or post-baseline VA assessment was available for 3 patients who were excluded from the FAS). The main reasons for early study withdrawal included the patient’s wish (*n* = 5), loss to follow-up (*n* = 2), and other reasons (*n* = 3).

In the XTEND Switzerland cohort, the mean ± SD age was 79.2 ± 7.4 years (range 59–91 years), and 31 patients (60.8%) were female (Table 1). At baseline, the mean ± SD BCVA was 64.9 ± 17.9 letters, and the mean ± SD CST was 402 ± 106 µm. A proactive T&E regimen was the intended regimen for most patients (*n* = 50).

### 3.2. Functional Outcomes

**Figure 2:**
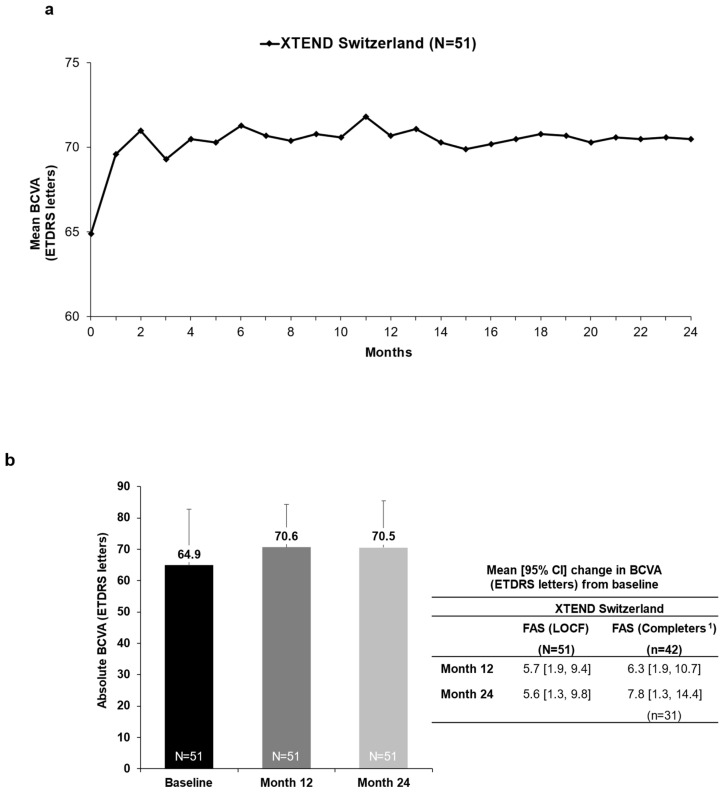
Visual acuity outcomes from baseline to Months 12 and 24 in patients with nAMD who were treated with IVT-AFL in routine clinical practice (FAS, LOCF). (**a**) Mean BCVA over 24 months. Mean BCVA data are reported based on the nearest VA assessments within monthly ± 15-day-visit windows. (**b**) BCVA (letters) from baseline to Month 12 and Month 24. Error bars denote SD. ^1^ FAS completers were defined as patients with valid visual acuity assessments at baseline and at Month 12 (360 ± 60 days). BCVA: best-corrected visual acuity; CI: confidence interval; ETDRS: Early Treatment Diabetic Retinopathy Study; FAS: full analysis set; IVT-AFL: intravitreal aflibercept; LOCF: last observation carried forward; nAMD: neovascular age-related macular degeneration; SD: standard deviation; VA: visual acuity.

**Figure 3:**
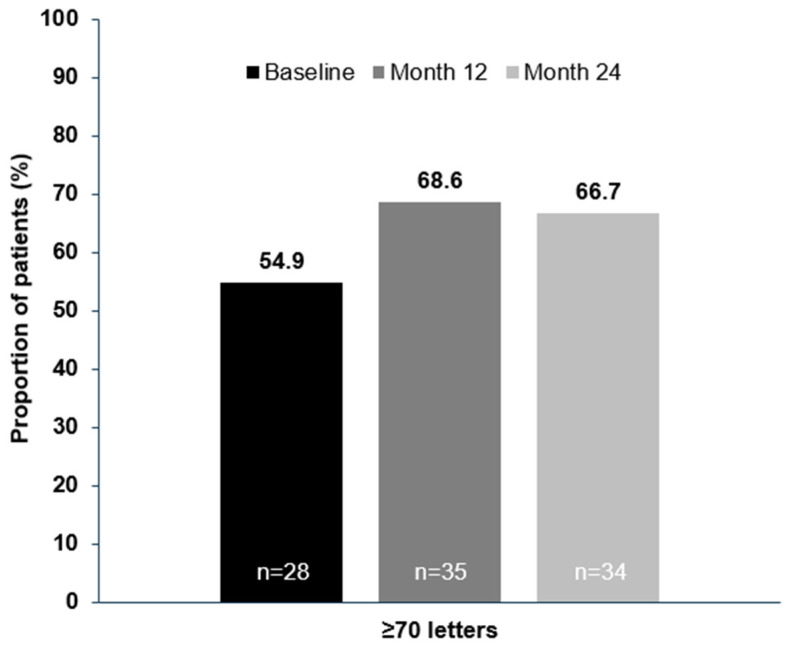
The proportion of patients achieving a VA letter score of ≥70 letters (Snellen equivalent of 20/40) by Month 12 and Month 24 (FAS, LOCF). FAS: full analysis set; LOCF: last observation carried forward; VA: visual acuity.

**Figure 4:**
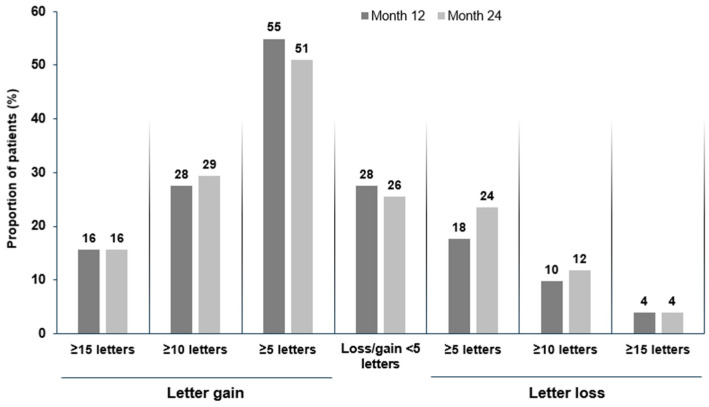
Letter gains and losses from baseline to Months 12 and 24 in treatment-naïve patients with nAMD following IVT-AFL treatment in routine clinical practice (FAS, LOCF). FAS: full analysis set; IVT-AFL: intravitreal aflibercept; LOCF: last observation carried forward; nAMD: neovascular age-related macular degeneration.

In the XTEND Switzerland FAS, patients’ mean (95% CI) change in BCVA from baseline was +5.7 (1.9, 9.4) letters at Month 12 and +5.6 (1.3, 9.8) letters at Month 24 (Figure 2a,b). In patients with a baseline VA of ≥70 letters, BCVA was generally maintained at ≥70 letters, with a mean (SD) increase of +1.0 (6.4) letter by Month 12 and +1.1 (6.3) letters by Month 24 (Supplementary Materials Figure S1). Overall, the proportion of patients with VA ≥ 70 letters (approximately 20/40 Snellen equivalent) in the studied eye increased from 54.9% (28/51) at baseline to 66.7% (34/51) by 24 months (Figure 3). Overall, 96.1% (49/51) of patients maintained their vision (≤15 letter loss) from baseline to Month 24 (Figure 4). The proportion of treatment-naïve patients who achieved ≥5-letter, ≥10-letter, and ≥15-letter VA gains by Month 24 were 51.0%, 29.4%, and 15.7%, respectively.

Of the FAS completers (*n* = 42), defined as patients with visual acuity assessments at baseline and at Month 12 (360 ± 60 days), the mean BCVA at baseline was 64.0 ± 18.9 letters. In this population, the mean (95% CI) change from the baseline at Month 12 was +6.3 (1.9, 10.7) letters (*n* = 42) and at Month 24 was +7.8 (1.3, 14.4) letters (*n* = 31) (Figure 2b).

### 3.3. Anatomic Outcomes

In the XTEND Switzerland FAS, the mean ± SD CST decreased from 402 ± 106 µm at baseline to 281 ± 56 µm at 12 months (mean [95% CI] change −125 [−161, −90] μm) and to 277 ± 56 µm at 24 months (mean [95% CI] change −127 [−162, −93] μm; Supplementary Materials Table S2 and Supplementary Materials Figure S2).

### 3.4. Treatment Pattern

For the XTEND Switzerland cohort, the mean ± SD time from diagnosis to the first IVT-AFL injection was 3.3 ± 3.7 days, and the mean ± SD time in the study (defined as the days between the first injection and last documented visit) was 20.4 ± 6.7 months (Table 2). The majority of IVT-AFL injections were received within the first 12 months of treatment, with a reduction in injection frequency observed thereafter. In total, patients received a mean ± SD of 6.5 ± 1.6 injections between baseline and Month 6; 9.5 ± 3.2 injections between baseline and Month 12; and 13.7 ± 6.0 injections between baseline and Month 24 (Table 2).

**Figure 5:**
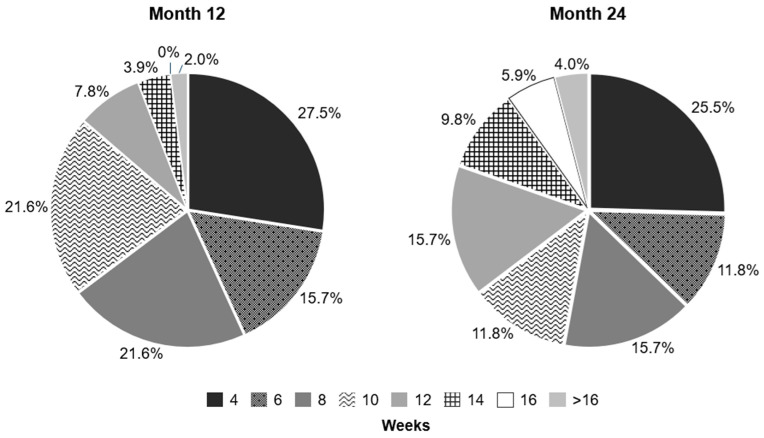
Last completed treatment interval by Months 12 and 24 (FAS, OC). Values may not add up to 100% due to rounding. FAS: full analysis set; OC: observed cases.

For Month 12, the last completed treatment interval was ≥8 weeks in 56.9% (29/51) of patients, ≥12 weeks in 13.7% (7/51) of patients, and ≥16 weeks in 2.0% (1/51) of patients. For Month 24, the last completed treatment interval was ≥8 weeks in 62.7% (32/51) of patients, ≥12 weeks in 35.3% (18/51) of patients, and ≥16 weeks in 9.8% (5/51) of patients (Figure 5).

### 3.5. Impact of COVID-19

All patients in the XTEND Switzerland cohort were enrolled during (or shortly before) the COVID-19 pandemic. Of the 51 patients in the FAS, 17 patients (33.3%) experienced ≥1 delayed injection due to the pandemic (“delay” is determined by the treating physician).

### 3.6. Safety

For patients in the SAS, TEAEs were reported in 37.0% (20/54) (Supplementary Materials Table S3). Drug-related ocular TEAEs were reported in three patients (5.6%: one case each of punctate keratitis, reduced visual acuity, and visual impairment). Each non-ocular TEAE occurred in a single patient. A total of 15 patients (27.8%) reported ocular TEAEs in either eye, while 12 patients (22.2%) reported ocular TEAEs in the studied eye; each ocular TEAE in the studied eye occurred in a single patient. No cases of intraocular inflammation were reported. There was one death reported in the XTEND Switzerland cohort (pulmonary embolism), although this was not deemed to be related to the study drug.

## 4. Discussion

XTEND is the largest real-world study to date in IVT-AFL-treated patients with nAMD. This 24-month analysis of the XTEND study demonstrates the effectiveness of IVT-AFL in patients with treatment-naïve nAMD in routine clinical practice in Switzerland.

In the XTEND Switzerland cohort, treatment-naïve patients with nAMD receiving IVT-AFL achieved clinically relevant functional and anatomic improvements by Month 12 that were maintained up to Month 24. The mean improvement in BCVA was +5.7 letters by Month 12 and +5.6 letters by Month 24. This change from the baseline is higher than that observed in the XTEND Global cohort (+2.3 letters by Month 24) [25] but lower than that in the VIEW RCT (+7.6 letters by Week 96) [7]; however, it is worth considering that the mean baseline BCVA in VIEW (54.0 letters) was lower than that in the XTEND Switzerland cohort (64.9 letters), which may have afforded patients in VIEW the scope for greater improvements in BCVA following treatment. The change in BCVA observed in the Switzerland cohort at Month 24 is comparable with the improvements of +6.0 and +6.6 letters observed in previous observational studies in Switzerland [20,22].

In this study, patients in the XTEND Switzerland cohort had a mean BCVA of 64.9 letters at baseline, with more than half (54.9%) of patients having a score of ≥70 letters. The mean BCVA at baseline was 10.6 letters higher than in the XTEND Global cohort (54.3 letters at baseline) [25] and similar to previous observational studies in Switzerland (59.8–63.8 letters) [20,22,24]. Even so, greater functional improvements were achieved by patients in the Switzerland cohort than by patients in the Global cohort, despite the potential ceiling effect of a high baseline BCVA. The proportion of patients with a BCVA ≥70 letters increased from 54.9% at baseline to 66.7% at Month 24. Importantly, patients in the Switzerland cohort who began treatment with a relatively high baseline BCVA (≥70 letters) generally maintained their BCVA ≥70 letters over the 24-month follow-up period.

Patients also achieved improvements in CST (−125 µm and −127 µm at Month 12 and Month 24, respectively). As with the change in BCVA, there was a greater improvement in CST in the Switzerland cohort than that observed in the overall cohort of patients from XTEND (−106 µm at Month 12 and −109 µm at Month 24) [25], and this change is similar to what was observed in a previous observational study in Switzerland (ASTERIA; −111 µm and −121 µm at Month 12 and Month 24, respectively) [20]. The baseline CST in the Switzerland cohort was marginally higher than the baseline CST of the Global XTEND cohort (402 μm compared to 374 μm) [25] but comparable to the baseline CST in the ASTERIA study in Switzerland (404 μm) [20]. The greater numerical improvement in BCVA and CST in the XTEND Switzerland cohort compared to the Global cohort may be, in part, attributed to the shorter time between the diagnosis and the first injection (Switzerland, 3.3 ± 3.7 days; Global, 37.2 ± 266.2 days). It has been previously suggested that a delay from diagnosis to treatment (leaving patients with longer-standing symptoms) may result in poorer visual gains and that the early treatment of nAMD with an anti-VEGF treatment could lead to a positive impact on visual outcomes [30].

In the XTEND Switzerland cohort, the majority of IVT-AFL injections were administered in the first 12 months. A mean ± SD of 9.5 ± 3.2 and 13.7 ± 6.0 injections were administered by Month 12 and Month 24, respectively, with an average of 4.2 injections administered from Month 12 to Month 24. The reduction in injection frequency observed after Month 12 is consistent with the XTEND Global cohort [25] and other observational studies of IVT-AFL in nAMD [18,19,20,31]. Overall, patients in the Switzerland XTEND cohort received more injections by both Month 12 and Month 24 compared with the Global cohort (mean ± SD of 7.7 ± 2.7 and 10.8 ± 5.0 injections by Month 12 and Month 24, respectively) [25], which may account for the greater functional and anatomic improvements observed in patients in Switzerland.

In the XTEND Switzerland cohort, the last completed treatment interval was ≥12 weeks in 13.7% (7/51) of patients at Month 12 and 35.3% (18/51) of patients at Month 24. The Month 24 values are similar to the XTEND Global cohort (37.7%) and the ASTERIA study in Switzerland (37%) [20], whereas the XTEND Global cohort observed more patients who had a final completed treatment interval of ≥12 weeks at Month 12 (35.6%) [25]. The greater proportion of patients with a shorter treatment interval in the Switzerland cohort, paired with the higher number of IVT-AFL injections received by Month 12 and 24 (9.5 ± 3.2 and 13.7 ± 6.0 injections by Month 12 and 24, respectively) and previous data published from the ASTERIA trial [20], may suggest that physicians in Switzerland adopted an “intensive treatment” approach to nAMD. In a post hoc analysis of the ARIES study, this approach was found to be an effective course of treatment and was associated with positive visual outcomes [32]. Moreover, patients in the Switzerland cohort had, on average, a short time between nAMD diagnosis and initial treatment (3.3 ± 3.7 days), suggesting that a “treat early, treat intense” approach may have contributed to the meaningful BCVA change at Month 24 despite the higher baseline BCVA observed in the Switzerland cohort compared to the Global cohort, although further analysis is required to confirm this.

Of note, 25.5% of patients in the XTEND Switzerland cohort had a final completed treatment interval of 4 weeks, and 35.3% had a final completed treatment interval of 12 weeks or higher at Month 24, highlighting the importance of tailoring treatment decisions to individual patient needs. T&E offers the possibility of tailoring treatment decisions to individual needs compared with fixed dosing regimens. In the XTEND Switzerland cohort, 50 patients were intended to follow a T&E approach. The analysis does not capture the final treatment regimen of each patient; however, the authors expect that a high proportion could have remained for T&E through to Month 24 based on their experience that T&E is the preferred proactive treatment in Switzerland and that approximately half of the patients had a final completed dosing interval of more than 8 weeks. Indeed, many findings from this analysis of patients from the Switzerland cohort should be contextualized with the baseline VA of patients and also with the Swiss healthcare system, which has the infrastructure and capacity to enable good access to treatment for most patients, with fewer restrictions on treatment, allowing for the early and intensive treatment of nAMD. The authors also acknowledge that due to the real-world nature of the XTEND study, the last completed treatment intervals recorded may have been influenced by factors such as missed injections or early discontinuation, which may limit the interpretation of these findings. Finally, the safety profile of IVT-AFL was consistent with prior observational studies over 24 months [7,33]. There were no cases of intraocular inflammation reported in the Switzerland cohort.

The strengths of XTEND include the prospective observational design, which is the first to be conducted in nAMD in Switzerland, and the long-term study duration. Observational studies provide a comprehensive understanding of treatment exposure, disease progression, disease burden, and long-term safety in routine clinical practice [14]. The data from XTEND also strengthen the evidence base for established therapy with aflibercept 2 mg to inform guidelines and clinical recommendations, especially in geographies where higher-dose anti-VEGF treatments are not authorized or indicated for nAMD. Real-world studies offer an opportunity to consider heterogenous populations and variability in populations, adherence, and treatment monitoring, which RCTs are not able to provide [34]. Country data such as these provide insights into practice patterns and clinical routines that are otherwise unable to be captured in larger studies. In Switzerland, treatment for nAMD begins promptly after diagnosis, and there are no restrictions on treatment frequency; thus, observational data on how the clinical practice and use of treatment regimens is impacted by the label change in aflibercept 2 mg over a maximum of 16 weeks are valuable. The insights provided by the Switzerland cohort analysis are different from those described in the publication of data from the UK cohort of XTEND, where vision gains were likely impacted by patients being of older age than those in the Global cohort alongside specific guidance on treatment regimens given by the Royal College of Ophthalmologists to UK ophthalmologists, and by the UK response to the COVID-19 pandemic [35]. Such important insights would have been difficult to obtain without the multicenter design of XTEND, allowing data collection in a variety of real-world settings. Conversely, there are limitations inherent to the observational study design. As clinical decisions were made based on physician discretion, the results from this small subgroup analysis, including 51 patients, may be highly variable. In addition, missing data due to loss to follow-up, as commonly experienced in real-world studies, can limit the interpretation of findings [36].

## 5. Conclusions

In the XTEND Switzerland cohort, IVT-AFL 2 mg proactive treatment, predominantly given as T&E, resulted in meaningful anatomic and functional improvements in treatment-naïve patients with nAMD across the 24-month period. Moreover, the treatment burden was reduced between Months 12 and 24, with extended treatment intervals observed at Month 12 and further extended to Month 24. Although decreasing injection frequency was observed throughout the study, BCVA gains were generally maintained over the 24 months, suggesting the long-term durability of IVT-AFL 2 mg clinical outcomes. These results indicate that, in real-world conditions, a proactive treatment regimen of IVT-AFL 2 mg can result in improved patient outcomes.

## Figures and Tables

**Table 1:** Patient baseline demographics and disease characteristics (FAS).

Characteristic	XTEND Switzerland
Number of patients (%)	51 (100.0)
Age, years	79.2 ± 7.4
Sex, *n* (%)	
Female	31 (60.8)
Race, *n* (%) ^1^	
European ancestry	41 (80.4)
Not reported	10 (19.6)
Mean BCVA, ETDRS letters	64.9 ± 17.9
Mean CST, µm	402 ± 106
BCVA letter score category, *n* (%)	
<35	3 (5.9)
≥35 to <70	20 (39.2)
≥70	28 (54.9)
Primary intended treatment regimen after initial monthly injections, *n* (%)	
Proactive treat-and-extend	50 (98.0)
Proactive fixed treatment	1 (2.0)

Values are mean ± SD unless otherwise stated. ^1^ Classified by the investigator or patient using fixed categories. BCVA: best-corrected visual acuity; CST: central subfield thickness; ETDRS: Early Treatment Diabetes Retinopathy Study; FAS: full analysis set; SD: standard deviation.

**Table 2:** The mean time from diagnosis to first IVT-AFL treatment, the mean time in the study, and the mean number of IVT-AFL treatments from baseline to Months 12 and 24 (FAS, OC).

	XTEND Switzerland (N = 51)
Mean time from diagnosis to first IVT-AFL treatment, days	3.3 ± 3.7
Mean time spent in the study, months	20.4 ± 6.7
IVT-AFL treatments	
BL to Month 6	6.5 ± 1.6
BL to Month 12	9.5 ± 3.2
BL to Month 24	13.7 ± 6.0

Values are mean ± SD. BL: baseline; FAS: full analysis set; IVT-AFL: intravitreal aflibercept; OC: observed cases; SD: standard deviation.

## Data Availability

The availability of the data underlying this publication will be determined later according to Bayer’s commitment to the European Federation of Pharmaceutical Industries and Associations/Pharmaceutical Research and Manufacturers of America “principles for responsible clinical trial data sharing”. This pertains to the scope, time point, and process of data access. As such, Bayer commits to sharing, upon request from qualified scientific and medical researchers, patient-level clinical trial data, study-level clinical trial data, and protocols from clinical trials in patients for medicines and indications approved in the United States (US) and European Union (EU) as necessary for conducting legitimate research. This applies to data on new medicines and indications that have been approved by the EU and US regulatory agencies on or after 1 January 2014. Interested researchers can use www.clinicalstudydatarequest.com to request access to anonymized patient-level data and supporting documents from clinical studies to conduct further research that can help advance medical science or improve patient care. Information on the Bayer criteria for listing studies and other relevant information is provided in the ‘Study sponsors’ section of the portal. Data access will be granted to anonymized patient-level data, protocols, and clinical study reports after approval by an independent scientific review panel. Bayer is not involved in the decisions made by the independent review panel. Bayer will take all necessary measures to ensure that patient privacy is safeguarded.

## Supplementary Materials

The following supporting information can be downloaded at https://www.mdpi.com/article/10.3390/jcm14072370/s1. Figure S1: Mean change in BCVA from baseline to Month 12 and Month 24 following IVT-AFL treatment in treatment-naïve patients with nAMD compared to baseline BCVA (FAS, LOCF); Figure S2: Mean change in CST measured by OCT (μm) from baseline to Month 12 and Month 24 in patients with treatment-naïve nAMD (FAS, LOCF); Table S1: Ethics approval boards; Table S2: Mean change in CST measured by OCT (μm) from baseline to Month 12 and Month 24 in patients with treatment-naïve nAMD (FAS, LOCF); Table S3: Safety overview culminated over 2 years (SAS).

## Congress Publication

This article is a revised and expanded version of a paper titled, “Two-year results from the Swiss cohort of a non-interventional study investigating real-world proactive dosing regimens with intravitreal aflibercept in patients with nAMD: XTEND study”, which was presented at Schweizerische Ophthalmologische Gesellschaft (SOG), Lausanne, Switzerland, 30 August–1 September 2023 [35].
