# Supplementary material for: Two-Year Switzerland Cohort Results from a Global Observational Study Investigating Proactive Dosing with Intravitreal Aflibercept 2 mg in Neovascular Age-Related Macular Degeneration
Source: J Clin Med. 2025 Mar 29;14(7):2370. doi: 10.3390/jcm14072370 (PMC11989833; doi:10.3390/jcm14072370)
Supplement: Supplementary file 1 [file jcm-14-02370-s001.zip › XTEND Swiss 2Y ms_suppl_v3.0_28Mar25.pdf]

SUPPLEMENTARY MATERIALS

Online Supplementary Figure S1 Mean change in BCVA from baseline to Month 12 and Month 24 following IVT-AFL treatment in treatment-naïve patients with nAMD by baseline BCVA (FAS, LOCF)

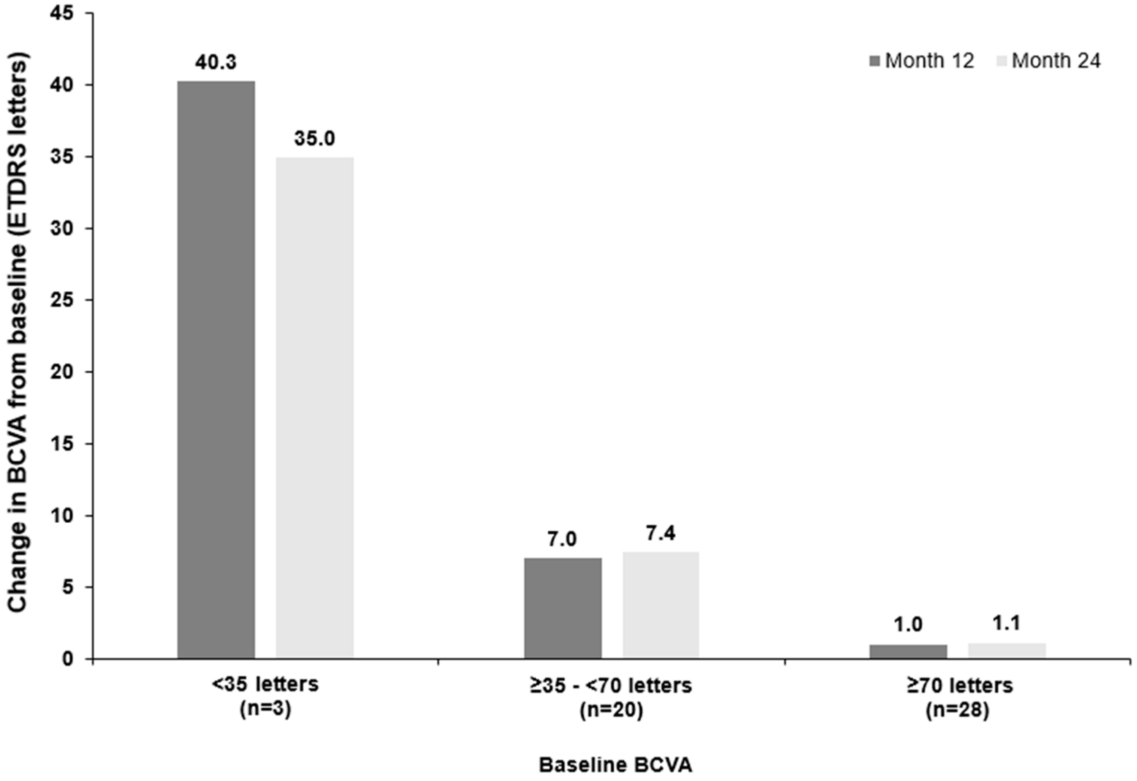

BCVA best-corrected visual acuity, *ETDRS* Early Treatment Diabetic Retinopathy Study, *FAS* full analysis set, *IVT-AFL* intravitreal aflibercept, *LOCF* last observation carried forward, *nAMD* neovascular age-related macular degeneration.

Online Supplementary Figure S2 Mean change in CST measured by OCT (μm) from baseline to Month 12 and Month 24 in patients with treatment-naïve nAMD (FAS, LOCF)

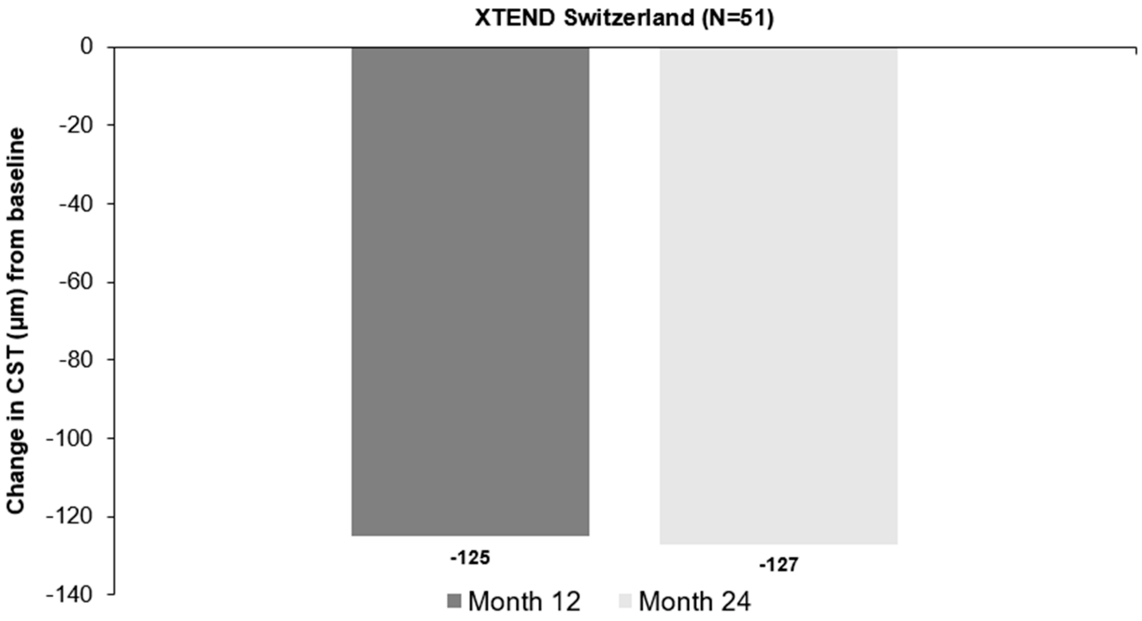

The mean CST change data are based on the nearest CST assessment within the ±60-day visit window of 360 days for Month 12 and 720 days for Month 24. *CST* central subfield thickness, *FAS* full analysis set, *LOCF* last observation carried forward, *nAMD* neovascular age-related macular degeneration, *OCT* optical coherence tomography.

Online Supplementary Table S1 Ethics approval boards

Country	Site number	Site name	Name of the ethics committee	Number/ID of the approvals
Switzerland	58001	Vista Klinik Binningen	Ethikkommission Nordwest- und Zentralschweiz (EKNZ)	2019-01573
Switzerland	58002	Universitätsspital Basel		
Switzerland	58003	Stadtspital Triemli		
Switzerland	58004	RétinElysée		
Switzerland	58005	Universitätsspital Zurich		
Switzerland	58006	Luzerner Kantonsspital		

Online Supplementary Table S2 Mean change in CST measured by OCT (μm) from baseline to Month 12 and Month 24 in patients with treatment-naïve nAMD (FAS, LOCF)

	XTEND Switzerland (N=51)
**Baseline**	
Mean CST	402±106
n (%)	49 (96.1)
**Month 12**	
Mean CST	281±56
Change from BL	–125 [–161, –90]
n (%)	50 (98.0)
**Month 24**	
Mean CST	277±56
Change from BL	–127 [–162, –93]
n (%)	50 (98.0)

Data are mean±SD and mean [95% CI] unless otherwise stated. The mean CST change data are based on the nearest CST assessment within the ±60-day visit window of 360 days for Month 12 and 720 days for Month 24. *BL* baseline, *CI* confidence interval, *CST* central subfield thickness, *FAS* full analysis set, *LOCF* last observation carried forward, *nAMD* neovascular age-related macular degeneration, *OCT* optical coherence tomography, *SD* standard deviation.

Online Supplementary Table S3 Safety overview cumulative up to 2 years (SAS)

Number of patients (%)	XTEND Switzerland (N=54)

**Any AE**	22 (40.7)
**Any TEAE**	20 (37.0)
Any non-ocular TEAE	9 (16.7)
Anal fissure	1 (1.9)
Cardiovascular disorder	1 (1.9)
COVID-19	1 (1.9)
Fall	1 (1.9)
Hyponatraemia	1 (1.9)
Hypovolaemia	1 (1.9)
Lung neoplasm malignant	1 (1.9)
Pneumonia	1 (1.9)
Pulmonary embolism [1]	1 (1.9)
Night sweats	1 (1.9)
Urinary tract infection	1 (1.9)
Any ocular TEAE	15 (27.8)
Any ocular TEAE in the study eye [2]	12 (22.2)
Blepharitis	1 (1.9)
Blindness transient	1 (1.9)
Cataract	1 (1.9)
Cataract operation	1 (1.9)
Chalazion	1 (1.9)
Conjunctivitis	1 (1.9)
Dry eye	1 (1.9)
Foreign body sensation in eyes	1 (1.9)
Lacrimation increased	1 (1.9)
nAMD [3]	1 (1.9)
Punctate keratitis	1 (1.9)
Retinal neovascularisation	1 (1.9)
Subretinal fluid	1 (1.9)
VA reduced	1 (1.9)
Visual impairment	1 (1.9)
Vitreous detachment	1 (1.9)
Any ocular TEAE in the fellow eye	7 (13.0)
Any drug-related ocular TEAE [4]	3 (5.6)
**Any serious TEAEs**	5 (9.3)
Any serious ocular TEAE	1 (1.9)
nAMD [3]	1 (1.9)
Any serious non-ocular TEAE [5]	4 (7.4)
Pneumonia	1 (1.9)
Fall	1 (1.9)
Lung neoplasm malignant	1 (1.9)
Pulmonary embolism [1]	1 (1.9)
Any serious drug-related TEAE	0

Values are n (%). [1] Resulted in death. [2] Two ocular TEAEs in the study eye are not yet coded. [3] Worsening of nAMD (as defined in the study protocol) in the study eye. [4] One case each of punctate keratitis, visual acuity reduced, and visual impairment. [5] One serious non-ocular TEAE is not yet coded. *AE* adverse event, *nAMD* neovascular age-related macular degeneration, *SAS* safety analysis set, *TEAE* treatment-emergent adverse event, *VA* visual acuity.
