# Supplementary material for: Two-Year Switzerland Cohort Results from a Global Observational Study Investigating Proactive Dosing with Intravitreal Aflibercept 2 mg in Neovascular Age-Related Macular Degeneration
Source: J Clin Med. 2025 Mar 29;14(7):2370. doi: 10.3390/jcm14072370 (PMC11989833; doi:10.3390/jcm14072370)
Supplement: Supplementary file 1 [file jcm-14-02370-s001.zip › XTEND Swiss 2Y ms_XTEND study collaborators_28Mar25.pdf]

SUPPLEMENTARY MATERIALS

XTEND study group

Collaborator	Study Country
Adam Booth	United Kingdom
Adisai Varadisai	Thailand
Adriana Correa	Colombia
Afsar Jafree	United Kingdom
Ahmad Sharara	United Kingdom
Aitor Eiras Fernández	Spain
Alain Donati	France
Alain Gregoire	Canada
Alban Comet	France
Alejandro Fonollosa Calduch	Spain
Alejandro Grondona	Argentina
Alice Degoumois	France
Alicia Esmeralda Traveset Maeso	Spain
Álvaro Manuel de Casas Fernández	Spain
Amer Omar	Canada
Amy Cohn	Australia
Amy Cohn	Australia
Ana González Gómez	Spain
Ana Sofía Delgado Fernández	Spain
Ana Sofía Delgado Fernández	Spain
Andreas Abou Taha	Denmark
Andreea Ionean	United Kingdom
Andres Lasave	Argentina
Andrew Chang	Australia
Ángela Gómez Moreno	Spain
Anik Desgroseilliers	Canada
Ann-Pascale Guagnini	Belgium
Anne Borlon	Belgium
Anne Briek	Colombia
Anne Linn Vatne Jacobsen	Norway
Anne Robinet	France
Antonio Manuel Soler García	Spain

Archana Airody	United Kingdom
Arif Samad	Canada
Asan Kochkorov	Switzerland
Aseema Misra	United Kingdom
Atitaya Apivatthakakul	Thailand
Aude Ambresin	Switzerland
Aude Couturier	France
AYoung Kim	South Korea
Baek-Lok Oh	South Korea
Bart Lafaut	Belgium
BEATRIZ DE LUIS EGUILEOR	Spain
BEATRIZ ENDO	Colombia
Belkacem Haouchine	France
Berit Feller Mortensen	Norway
BoHee Kim	South Korea
Boris Rysanek	France
Bushra Mushtaq	United Kingdom
Candelaria Grondona	Argentina
Carlos Perálvarez Conde	Spain
Carlos Plaza Laguardia	Spain
Carmela Maceri	France
Carmen Alba Linero	Spain
Carmen Alba Linero	Spain
Carmen Jurjo	Spain
Carolina Arruabarrena Sánchez	Spain
Carolina Sardi	Colombia
Caroline Tolou	France
Catherine Creuzot-Garcher	France
Catherine Favard	France
Catherine Français	France
Cecilia Goycoa	Argentina
Céline Faure	France
Cengiz Türksever	Switzerland
Chafik KEILANI	France
Chang Ki Yoon	South Korea
Chaoyi Qu	China
Christian Prünte	Switzerland
Christian Svendsen	Norway
Christos Zoumpeloulis	Sweden
Chul Hee Lee	South Korea
ChulGu Kim	South Korea
Chunling Lei	China
Claire Scemama-Timsit	France
Clare Bailey	United Kingdom
Claudia Acosta	Colombia
Claudia Carvajal	Colombia

Claudio Furino	Italy
Colin Jones	United Kingdom
Coraline Calci	Switzerland
Corinne Dot	France
CRISTINA PEREZ CASASECA	Spain
CRISTINA PEREZ CASASECA	Spain
Cuiyun Yin	China
Cynthia Qian	Canada
Cynthia Santiago	United Kingdom
Cyril Dutheil	France
Daniel Barthelmes	Switzerland
Daniele Veritti	Italy
David Keegan	Ireland
David Rodríguez Feijoo	Spain
David Sayag	France
Deepali Varma	United Kingdom
Direk Patikulsila	Thailand
DongGeun Park	South Korea
DongHyun Lee	South Korea
DongWon Lee	South Korea
DongYun Yeon	South Korea
Dr.Thanapong Somkijrungroj	Thailand
Elin Benthin	Sweden
Els Mangelschots	Belgium
Emilie Agard	France
Emilio José Cebrián Rosado	Spain
Emily Fletcher	United Kingdom
Emmanuel Ankamah	Ireland
Enrico Peiretti	Italy
Éric Souied	France
Erlend Hoven	Norway
Eugene Ng	Ireland
Eugenia Gijón Vega	Spain
Eun Kyoung Lee	South Korea
Eunjung Choi	South Korea
EunYoung Choi	South Korea
Evangelos Minos	United Kingdom
Eve Rousseau	France
Evelyne Mulliez	Belgium
Facundo Urbinati	Spain
Facundo Urbinati	Spain
Felipe Mauricio Costales Mier	Spain
Filippo Tatti	Italy
Flor Gómez	Colombia
Flore De Bats	France
Florence Coscas	France

Francesca AMOROSO	France
Francesco Maria Bandello	Italy
Francisco Arango	Colombia
Francisco Berrero Sojo	Spain
Francisco José Barrero Sojo	Spain
Francisco Rodriguez	Colombia
Frédéric Chiambaretta	France
Frédéric Matonti	France
Gabriella De Salvo	United Kingdom
GaHyung Ryu	South Korea
Gary Yau	Canada
Geeta Menon	United Kingdom
Georgios Tsiropoulos	Switzerland
Gerald Liew	Australia
GeunWoo Lee	South Korea
Ghassan Cordahi	Canada
Ghislaine Traber	Switzerland
Giorgio Enrico Bravetti	Switzerland
Patrizia Busatto	Italy
Gisele Finocchio	Argentina
Giuseppe Fasolino	Belgium
Gonzaga Garay Aramburu	Spain
Guang'ming WAN	China
Gustavo Fernández-Baca Vaca	Spain
Gustavo Fernández-Baca Vaca	Spain
Gwyn Williams	United Kingdom
Haiyan Wang	China
Hamza Abdou	United Kingdom
Haralabos Eleftheriadis	United Kingdom
Harbhajan arora	United Kingdom
Hassiba Oubraham-Mebroukine	France
HeeYoon Cho	South Korea
Heleen Nicolai	Belgium
Helena Zimmermann	Switzerland
Hilde Taraldlien	Norway
Hildegard Piñeros	Colombia
Henrik Vorum	Denmark
Hong DAI	China
Hong Yan	China
HunJin Choi	South Korea
Hussam El Chehab	France
Hyeong Gon Yu	South Korea
HyeRim Choe	South Korea
HyungBin Lim	South Korea
HyunGoo Kang	South Korea

Ian Pearce	United Kingdom
Iksoo Byon	South Korea
Inger Westborg	Sweden
Inmaculada Lozano Escobar	Spain
Ioanis Papasavvas	Switzerland
IRATI HURTADO PRIETO	Spain
Iratxe Zabalza Aguirrezabala	Spain
IRENE HERRERO DIEZ	Spain
Isabel María Baquero Aranda	Spain
Isabelle Sillaire-Houtmann	France
Ivana Huzevkova	Sweden
Ivonne Charcán Laskibar	Spain
Jad Akesbi	France
Jae hui Kim	South Korea
Jae hui Kim	South Korea
Jae Yul Hwang	South Korea
JaeJung Lee	South Korea
Jagjit Gilhotra	Australia
James Muecke	Australia
James Talks	United Kingdom
Janejit Choovuthayakorn	Thailand
Javier Buendia	Colombia
Javier José Aguayo Álvarez	Spain
Jean-Antoine Pournaras	Switzerland
Jean-François Korobelnik	France
Jenny Berglund	Sweden
Jesús Zarallo Gallardo	Spain
JiEun Lee	South Korea
Jignesh Patel	United Kingdom
JiHun Song	South Korea
Jin Cho	South Korea
Jin Yao	China
Joël Gambrelle	France
Joël Uzzan	France
John Conrath	France
Joke Ruys	Belgium
Jonas Van Den Heurck	Belgium
Jong-uk Lee	South Korea
Joon Hyung Kim	South Korea
José Antonio Sánchez Aparicio	Spain
José María Calandria Amigueti	Spain
Juan Arias	Colombia
Juan Sanchez	Colombia
Juan Pablo Francos	Argentina
Julie Jacob	Belgium
Junwon Lee	South Korea

Kang Li	China
Karim Atmani	France
Katerina Ivanova	United Kingdom
Katja Hatz	Switzerland
Kichawatt Kullavanijhya	Thailand
Knud Beier Pedersen	Denmark
Konstantinos Niloakopoulos	Switzerland
Kristina Holm	Sweden
Kyu Hyung Park	South Korea
Kyung Jun Choi	South Korea
Laura Hoffmann	Switzerland
Laura Monje Fernández	Spain
Laura Monje Fernández	Spain
Laura Moschetta	Belgium
LAURA RODRIGUEZ GARCIA	Spain
Laurent Kodjikian	France
Laurent Lalonde	Canada
Lena Giralt Peret	Spain
Li-Ping Chow	Australia
Linda Neumann	Denmark
Lioi Susanna	Italy
Lisa Kelly	United Kingdom
LLUIS PEREZ MAÑA	Spain
Lorenzo Mangoni	Italy
Louis-Pierre Gauvin Meunier	Canada
Lourdes Grassi	Argentina
Luc Van Os	Belgium
Madeleine Kanku	Switzerland
Maialen Aldazábal Echeveste	Spain
Mandeep Bindra	United Kingdom
Manju Chandran	United Kingdom
Manuel Raiz	Argentina
Marc Saab	Canada
Marcel Dominguez	France
María Ángeles López-Egea Bueno	Spain
María Arantzazu Larrauri Arana	Spain
María Concepción Martínez Antón	Spain
María del Carmen López Quero	Spain
María Lourdes Macías Molinero	Spain
Maria Arango	Colombia
María Rosario Cobo Soriano	Spain
Maria Tyrberg	Sweden
Maria Zejmo	Sweden

Marie-Bénédicte Rougier	France
Marie-Noelle Delyfer	France
Marion Silvia Schroeder	Sweden
Mark Cahill	Ireland
Martin Schmid	Switzerland
Maté Streho	France
Matthias Becker	Switzerland
Mercedes Rodríguez	Argentina
Micaela Acosta	Argentina
Michael Brent	Canada
Michael Mills	Canada
Michael Spangsberg	Denmark
Michail Katzakis	United Kingdom
Michel Van Lint	Belgium
Michele Clerici	Switzerland
Mikael Thommassen Neset	Norway
Milagros Grondona	Argentina
Min Sagong	South Korea
Min-Su Kim	South Korea
Minh-Huyen Nghiem-Buffet	France
Mirinae Kim	South Korea
Monica Loevestam Adrian	Sweden
Monica Varano	Italy
MoonJeong Choi	South Korea
MYRIAN HERNANDEZ	Colombia
Naeem Iqbal	United Kingdom
Nathalie Puche	France
Nawat Watanachai	Thailand
Nerea Martínez Alday	Spain
Niro Narendran	United Kingdom
Nuria María Gajate Paniagua	Spain
Olav Maepea	Sweden
Óscar Ruiz Moreno	Spain
Pablo Álvarez Ramos	Spain
Pablo Coccaro	Argentina
Paola Giorno	Italy
Paolo Lanzetta	Italy
Paradee Kunavisarut	Thailand
Parravano Cristina	Italy
Patricia Gutiérrez Castaño	Spain
Patricia Gutiérrez Castaño	Spain
Paul Mitchell	Australia
Paula Salgado	Argentina
Pear Pongsachareonnont	Thailand
Peggy Guerrero	Colombia
Penelope Allen	Australia
Peng Yan	Canada
Pierre Blaise	Belgium

Pierre Gascon	France
Pierre Lepage-Létourneau	Canada
Pierre-Raphael Rothschild	France
Praveen Patel	United Kingdom
Qingshan Chen	China
Quresh Mohamed	United Kingdom
RaeYoung Kim	South Korea
Rafael Montejano Milner	Spain
Ramón Espinet Badía	Spain
Richard Gale	United Kingdom
Richard Thornton	United Kingdom
Robert Buttery	Australia
Roberto Martínez Díaz	Spain
Rosie Dawkins	Australia
Rossella Anzidei	United Kingdom
Rune Holmbjoern	Denmark
Ruxandra Hera	France
Salomon Cohen	France
Sara Touhami	France
Sarah Tick	France
Sarra Gattoussi	France
Sébastien Gagné	Canada
Sebastien Olivier	Canada
Seongyong Jeong	
Seung Min Lee	South Korea
SeungKwon Choi	South Korea
SeWoong Kang	South Korea
Shahrnaz Izadi	United Kingdom
Shane Durkin	Australia
Shenzhi Liang	China
Shuo Zhao	China
Simon Morgan	United Kingdom
Simon Rothenbühler	Switzerland
Sonia Astorga Moreno	Spain
Sonia Scaf	Colombia
Sophie Bonnin	France
Sophie Bonnin	France
Soren Kromann Abildgaard	Denmark
Stéphanie Baillif	France
Sturla Sandoy	Norway
Su Zhang	China
SungWho Park	South Korea
SunHo Park	South Korea
Suresh Thulasidharan	United Kingdom
SUSANA MEIJIDE DE LA FUENTE	Spain
Suthasinee Sinawat	Thailand
Tamara Zompa	Argentina

Tamara Zompa	Argentina
Tanya Moutray	United Kingdom
Teresa Diago Sempere	Spain
Thomas Bording Adams	Denmark
Thomas Sheidow	Canada
Thuss Sanguansak	Thailand
Timothy Hamann	Switzerland
Turgut Ferhat	Switzerland
UnChul Park	South Korea
Valentina Carta	Italy
Valentina Sarao	Italy
Valérie Krivosic	France
Ann Vanderschueren	Belgium
Varun Chaudhary	Canada
Vasileia Chatzistergiou	Switzerland
Vasileios Konidaris	United Kingdom
Victoria Van Renterghem	Belgium
Vincent Gualino	France
Vinzenz Vadasz	Switzerland
Violaine Caillaux-Legou	France
Vita Dingerkus	Switzerland
Viviane Guignard	Switzerland
Vladimir Poposki Hamamdjieva	Spain
Voraporn Chaikitmongkol	Thailand
Wipada Laovirojjanakul	Thailand
Won-bong Jang	South Korea
WooHyuk Lee	South Korea
WooKyung Park	South Korea
Xiangyan Wang	China
Xiaobing Yu	China
Xiaopei Zhang	China
Xizhen Wang	China
Yacine Ailem	France
Yanchun Zhang	China
Yingyi LU	China
YongIl Shin	South Korea
YongKyun Shin	South Korea
YongUn Shin	South Korea
Yooyoung Jeon	South Korea
YoungGun Park	South Korea
YoungHoon Park	South Korea
YoungJoon Jo	South Korea
Youxin Chen	China
Zaida Vega López	Spain
Zakariya Jarrar	United Kingdom
zhengpei zhang	China
Zoi Paparrizou	Sweden

Zuriñe del Barrio López de Ipiña	Spain
